# Supplementary material for: Two is better than one: Social rewards from two agents enhance offline improvements in motor skills more than single agent
Source: PLoS One. 2020 Nov 4;15(11):e0240622. doi: 10.1371/journal.pone.0240622 (PMC7641341; doi:10.1371/journal.pone.0240622)
Supplement: S2 File — (PDF) [file pone.0240622.s002.pdf]

Do Social Rewards from Robots Enhance Offline Improvements in Motor Skills?

Soto Okumura[1,2(✉)], Mitsuhiko Kimoto[1,2], Masahiro Shiomi[1], Takamasa Iio[1,3], Katsunori Shimohara[2], and Norihiro Hagita[1]

[1] ATR-IRC, Kyoto, Japan
okumura2016@sil.doshisha.ac.jp
[2] Doshisha University, Kyoto, Japan
[3] Osaka University, Osaka, Japan

Abstract. In human-human interaction, praise is a fundamental social reward. Praise provides many benefits to people who receive it, such as positive feelings and improved motivation as well as on specific skills and performances. In this paper, we investigate whether praise from two robots influences offline improvements on the motor skills in a manner that resembles praise from humans. We conducted an experiment with 27 participants to investigate the relationships between the effects of social rewards and the number of robots, by using a sequential finger-tapping task which is used in a similar past research work. Our experiment results showed that people who received praise from two robots showed significantly better offline improvements on the motor skill improvement than people who did not receive such praise, but there is no significant differences between two robots and one robot. Moreover, questionnaire results showed higher degree of satisfaction and confidence towards robots' speech sentences when the robots provided praises.

Keywords: Praise · Social rewards · Human-robot interaction · Multiple robots

1 Introduction

Praise is one fundamental social reward for human beings [1]. It provides various positive effects on people's mental states by boosting self-efficacy [2], improving motivation [3, 4], raising academic self-concept [5], and promoting pleasurable emotions [6]. Such positive effects on mental states improve people's skills and performances: simple go/no-go task performances [4], on-task behavior (e.g., students are engaged in activity which is being facilitated by teacher) [5], academic performances such as math skills, [7, 8] and motor skills such as mirror-tracing task [9, 10].

Since people perceive agent systems like computer-graphics agents and robots as social beings [11–13], social rewards from such agents also positively affect people's mental states and performance improvements: creating friendly feelings [14, 15], motivation improvement [16], and increasing of task performances such as game-based tasks, such as readability testing or puzzles [17, 18].

© Springer International Publishing AG 2017
A. Kheddar et al. (Eds.): ICSR 2017, LNAI 10652, pp. 32–41, 2017.
https://doi.org/10.1007/978-3-319-70022-9_4

However, even if these works manifested the effectiveness of social rewards from robot systems for improvements in just a short-term (e.g. within a day) performance, the effectiveness for a long-term improvements retention (e.g., more than one day) remain uninvestigated. Such long-term skill consolidation process is essential for human skill formation [19, 20], therefore if praise from robots influences such skill consolidation process and offline improvement (which is a form of consolidation, one of learning processes related to the amount of time spent [21, 22]) on the motor skill as directly as human praise [10], that knowledge would contribute to the design of human-robot interaction by focusing on long-term performance improvements.

In this study, we investigate the effects of praise from a robot system toward offline improvements on the motor skills. We conducted an experiment where our system either gave or didn't give praise to participants during tasks. Our experiments had two stages: on the first day the participants were praised after they completed tasks, and on the second day they re-performed tasks without praise.

We are also interested in the possibilities of using two robots for praises (Fig. 1). Because in human science literature, the number of others increases such social influences as social facilitation/loafing [23, 24], and performance such as creativity [25–27]. Using two robots changes the impressions and the behaviors of the interacting partners [28–30]. Thus, we hypothesize that the number of robots will influence the praise effects. In this study, we address the following research questions:

– Does praise from a robot influence offline improvements on the motor skills?
– Does the number of robots increase the effects of social rewards?

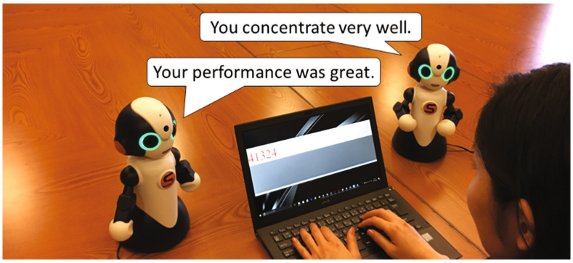

Fig. 1. Two robots praise a participant

2 System Overview

Figure 2 shows an overview of our system that consists of the following components: a robot (Fig. 3) and a GUI (Fig. 4). Participants perform training tasks through GUI, which sends start/end signals to the robots to control the timing of their speeches.

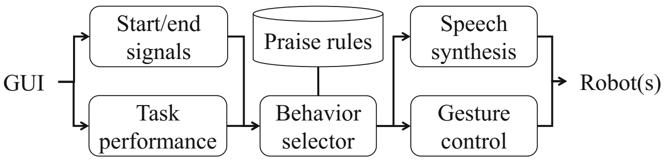

Fig. 2. System overview

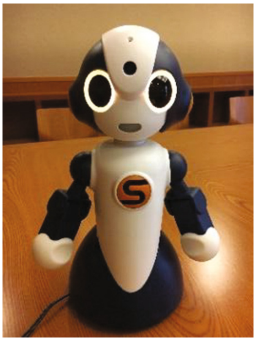

Fig. 3. Sota

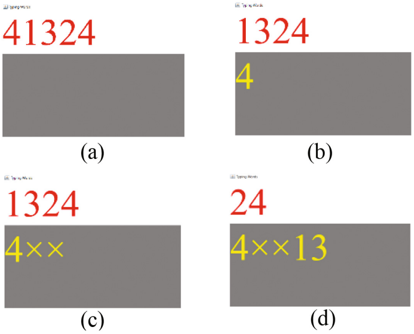

Fig. 4. GUI

2.1 Training Task Design to Investigate Offline Improvement of Motor Skill

In this study, we employed a task that resembles past research work [10], i.e., a sequential finger-tapping task, where participants repeatedly press four numeric keys (1, 2, 3, 4) on a standard computer keyboard as quickly and as accurately as possible for 30-seconds periods. This task is commonly used to investigate the offline improvements on the motor task in past studies therefore we also used the task for our study [10, 20, 31, 32]. Similar to thesm, the tapping sequence was common to all participants and trials; we used the following five number sequence in all tasks: 4-1-3-2-4.

To investigate the consolidation processes about the offline improvement on the motor skill, the participants joined two days experiments. They executed the task 12 time at the first day, and they executed the task five time at the second day. In the past study, the participants did not know whether they execute the same task at the second day, therefore we followed the same procedure and task design for our study.

2.2 GUI

Based on consideration of the training task, we developed a GUI (Fig. 4) that shows numeric numbers based on the pre-defined sequence by using Java (Fig. 4-a). During the tasks, if the participant correctly pressed the displayed number, it disappeared from

the computer screen's upper part and moves to its bottom part (Fig. 4-b, "4" was pressed). If the participant made a typing mistake, a cross appeared at the bottom part (Fig. 4-c, where the participant made two typing mistakes). If the participant correctly pressed the displayed numbers, they moved to the bottom part again (Fig. 4-d, "1" and "3" were pressed after two mistakes). At the end of each trial, both the numbers of correctly and incorrectly pressed keys are displayed and these data are recorded for analysis. The start/end times are sent to the robot by a network.

2.3 Robot

Figure 3 shows Sota, an interactive humanoid robot characterized by its humanlike physical expressions. Sota has eight DOFs: three in its head, one for its shoulders, one for its elbows, and one for its base. It is 28 cm tall, 140 mm wide, 160 mm deep, and weights 763 g. It is equipped with voice synthesis and network functions. While speaking, the robot gazes at the participant and slightly moves its arms.

2.4 Details of Praise Comments

Similar to the past study [10], we designed the robot to speak to the participant after each trial. To avoid skeptical feelings fueled by continuous praise and keep relatively consistent praises, we mixed pre-defined praise comments, neutral comments, and praise comments based on their performance in a specific rule as shown in Table 1.

For the pre-defined praise comments, we prepared seven comments that consist of two sentences, both of which include praise: "your performance is very good, especially if this is your first trial," "your typing speed seems faster than in the previous trial," and so on. We did not use the same sentence twice during the same day.

For neutral comments, we also prepared 12 comments that consist of two sentences. In the neutral comments, neither sentence included praise; 12 sentences consists of the current trial number and the rest amount of trials, e.g., "this completes the seven trial, the remaining trial is five", and two sentences consists of the current trial number and averaged performance, e.g., "this completes the third trial, your performance resembles that of other participants."

For praise comments based on performance, we prepared a rule-based selection algorithm. We used three features to decide the praise comments into six categories: correctly typed numbers, incorrectly typed numbers, and the total typed numbers (Table 2). There are 12 comments in total: two comments for six categories for each. These comments are used in the middle of tasks to avoid skeptical feelings using continuously pre-defined praise comments, i.e., after 4th trial and 6th trial; if the same

Table 1. Sequence of praise comments

Order	Behavior categories
1	Pre-defined praise behavior
2	Pre-defined praise behavior
3	Neutral comment
4	Praise behavior based on performance
5	Pre-defined praise behavior
6	Praise behavior based on performance
7	Neutral comment
8	Pre-defined praise behavior
9	Pre-defined praise behavior
10	Pre-defined praise behavior
11	Pre-defined praise behavior
12	Pre-defined praise behavior

Table 2. Pseudocode of praise selection

```
// Praise behavior selection algorithm

if (correct_typing > before_correct_typing)
        praise(increasing_correct_typing)
else if (miss_typing==0)
        praise(no_error)
else if (correct_typing > threshold_correct_typing)
        praise(certain_correct_typing)
else if (miss_typing < before_miss_typing)
        praise(certain_error_typing)
else if (total_typing >= before_total_typing)
        praise(maintaining_performance)
else
        praise(attitude_for_training)
```

category is selected in both timings, different comments are used in each timing. The following are the details of the comments in each category:

Increase of correctly typed numbers: the robot praises the number of correctly typed words if that amount exceeds maximum number at this point: “since you typed more numbers correctly, your typing speed is becoming faster.”
No errors: the robot praises when the participant did not incorrectly typed: “since you made no mistakes, your typing is really accurate.”
Certain correct typing: the robot praises the number of correctly typed numbers if it exceeds a threshold (in this study it was 150): “your typing record is good since the number of correctly typed numbers is above average.”
Certain error typing: the robot praises fewer errors if they are less than minimum number at this point: “since you’ve decreased your typing errors, your typing is becoming more correct.”
Maintaining performance: the robot praises a performance that maintained the training task if the amount of correctly typed words did not exceed maximum number at this point, the amount of errors did not lower from minimum number at this point, and the total amount of typed numbers exceed the last task. In this case, since the performance are neither better nor bad, the robot praises the person’s concentration: “you are maintaining your performance and concentrating.”
Attitude for training: the robot praises the attitude to the training task when the participant’s performance was not better and when the total amount of typing did not exceeded the last task value. In this case, since the performance did not actually improve, the robot praised the attitude: “since your typing performance seems to have stabilized, your performance will probably increase.”

3 Experiment

3.1 Hypothesis and Predictions

We assume that people's offline improvements on the motor skills will increase if they get praise from a robot. Past research in human science literature showed that social rewards enhance the offline improvement on the motor skills of people [10] and produce positive effects (without offline improvement on the motor skill improvement) of social rewards not only from humans but also non-humans, i.e., agent systems [16–18].

Moreover, we assume that the number of robots will increase the effect of praise, i.e., more enhancing offline improvement on the motor skills of people. Past research works showed that many people [23, 24] and agents [28–30] have stronger social influences on people and change their behaviors and/or impressions. Based on these considerations, we made the following predictions:

Prediction 1: Offline improvement on the motor skills will be enhanced more when people are praised by one robot than people who are not praised.
Prediction 2: Offline improvement on the motor skills will be enhanced more when people are praised by two robots than people who are not praised.
Prediction 3: Offline improvement on the motor skills will be enhanced more when people are praised by two robots than people who are praised by one robot.

3.2 Participants

Twenty seven university students (12 women and 15 men who averaged 20.9 years of age, S.D: 0.87) participated.

3.3 Environment

We conducted it in a laboratory room. We installed one or two robots and a PC on a desk. We recorded video and audio data for analysis.

3.4 Condition

The study had a between-participants design with the following three conditions: one robot, two robots, and no-praise. We randomly assigned each participant to one of three conditions, by keeping gender ratios between them.

One robot: In this condition, we used one robot (Fig. 5). The comment of the robot after each task is decided based on the rules of Tables 1 and 2.

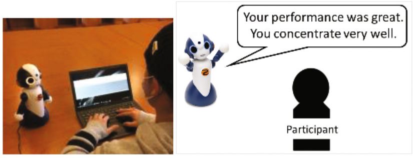

Fig. 5. One-robot condition

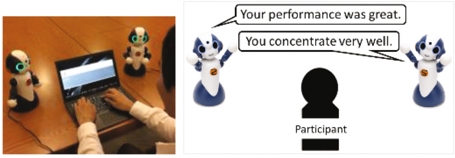

Fig. 6. Two-robot condition

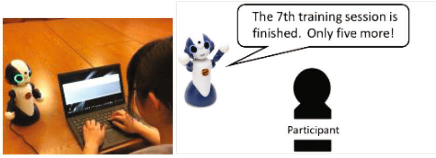

Fig. 7. No-praise condition

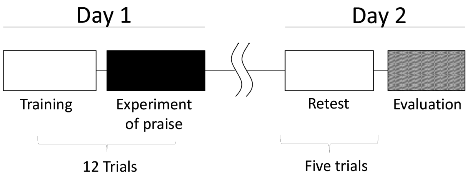

Fig. 8. Experiment procedure

Two robots: In this condition, we used two robots (Fig. 6). The comment of the robot after each task is decided based on the rules of Tables 1 and 2, too. All comments which include two sentences were separately made by two robots. The speaking sequence was the same; in this study, the robot to the left of the computer spoke first.

No-praise: In this condition, we used one robot. Unlike the one-robot condition, the robot used 12 neutral comments (Fig. 7). Thus, it only used neutral comments after each task, instead of pre-defined praise comments and those based on performances.

3.5 Procedure

We followed a similar procedure from past research [10], i.e., an experiment that was conducted on two subsequent days. Before the experiment on the first day, the participants were given a brief description of our experiment's procedure.

On day 1, participants were trained with sequential finger-tapping tasks on 12 trials. After starting the experiment, the robot greets the participant and explains its role as a MC. Next it asks the participants to start the training task and explains that the experiment will end after 12 trials. After each training task, the robot(s) make pre-defined sentences and rules, as described above (Fig. 8, left). After making its comments, the robot suggests taking a 20-second break before starting the next trial. At the end of day 1 experiment, we told the participants to do different kind of task at tomorrow, to minimize the possibility that they might physically or mentally practice the training sequence prior to the retest, based on the conclusion of the past study [10].

On day 2, the participants again participated to the sequential finger-tapping task on five trials (Fig. 8, right), different from the explanations of the last day. On day 2, the robot(s) did not praise the participants in all conditions, only making neutral comments. We used the same tapping sequence for all tasks in the both days.

3.6 Measurement

We measured the offline improvement on the motor skills by comparing the performance of the finger-tapping training for two continuous days. Because the offline performance improvement was defined as the percent increase in average performance from the last three trials during training on day 1 compared with the first three retest trials on day 2 in the sequential finger-tapping task [10, 32], we measured them.

4 Results

4.1 Manipulation Check

Figure 9 shows the mean performances during the last three training trials on day 1 and the first three retest trials on day 2. For a manipulation check, we investigated whether the performances of each group are significantly different or not. We conducted an ANOVA for the mean performance during the last three training trials on day 1, and the result did not show significant differences between them ($F(2, 24) = 0.64$, $p = 0.938$, *partial* $\eta 2 = 0.05$). Therefore, the mean performances during the last three training trials on day 1 were not significantly different between conditions.

4.2 Verification of Predictions

Figure 10 shows the offline improvement of each condition, which is the percent increase from the mean performance on day 1 to day 2. We conducted an ANOVA and its results showed significant differences among the three conditions ($F(2, 24) = 5.376$, $p = 0.012$, *partial* $\eta 2 = 0.309$). Multiple comparisons with the Bonferroni method revealed a significant difference: *two* > *no-praise* ($p = .010$). There was no significance between *two* and *one* ($p = .289$) or *one* and *no-praise* ($p = .406$) Thus, predication 2 was supported, but predictions 1 and 3 were not.

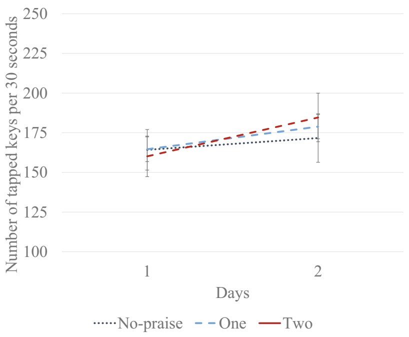

Fig. 9. Mean performance

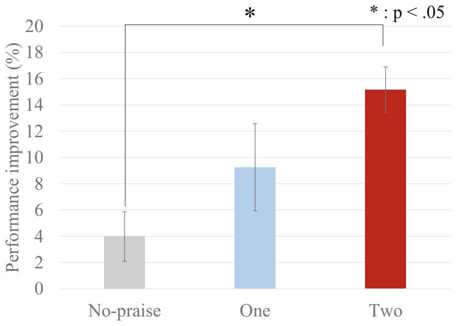

Fig. 10. Offline improvements

5 Discussion

Our experiment results revealed that praise from two robots positively influenced their offline improvements on the motor skill improvement, although praise from one robot did not show significant improvement. The results might suggest that the number of robots increases the effects of social rewards, because the effects of social rewards from one robot did not significantly improve the performance compared to non-praise condition. One interesting future work is to investigate whether praises from two persons or agents increase performances more than one person or agent.

Since our experiment was conducted with our robot system and its specific settings, knowledge generality is limited. We cannot confirm whether our finding about using two robots for praising can be applied to other contexts or different tasks. Another consideration is the naturalness of praise in more complex situations. In this research work, since the robots praised the participants regardless of their actual performance in some parts of the trials, directly applying such other situations as training many people together is difficult. Also, the praise selection algorithms are implemented heuristically. However, we believe that our setting is adequate to offer knowledge for readers who are interested in social reward effects from two robots.

6 Conclusion

We investigated the effects of social rewards from two robots for offline improvements on the motor skill improvement. We prepared praise comments for two social robots and finger-tapping tasks by following past research, which also investigated the effects of social rewards. Our experimental results showed that people who were praised by two robots showed higher motor performance than people who did not receive such praise. On the other hand, the performances were not different between people who received praise from two robots or one robot, and between people who received praise from one robot and who did not receive such praise.

Acknowledgements. This research work was supported by JSPS KAKENHI Grant Numbers JP15H05322 and JP16K12505.

References

1. Buss, A.H.: Social Behavior and Personality (Psychology Revivals). Routledge, Evanston (2014)
2. Bandura, A.: Self-efficacy: toward a unifying theory of behavioral change. Psychol. Rev. **84**(2), 191 (1977)
3. Ames, C.: Motivation: What teachers need to know. Teachers Coll. Rec. **91**(3), 409–421 (1990)
4. Kohls, G., Peltzer, J., Herpertz-Dahlmann, B., Konrad, K.: Differential effects of social and non-social reward on response inhibition in children and adolescents. Dev. Sci. **12**(4), 614–625 (2009)
5. Chalk, K., Bizo, L.A.: Specific praise improves on-task behaviour and numeracy enjoyment: a study of year four pupils engaged in the numeracy hour. Educ. Psychol. Pract. **20**(4), 335–351 (2004)
6. Aliyev, R., Karakus, M., Ulus, H.: Teachers' verbal cues that cause students to feel various emotions. Anthropologist **16**(1–2), 263–272 (2013)
7. Henderlong, J., Lepper, M.R.: The effects of praise on children's intrinsic motivation: a review and synthesis. Psychol. Bull. **128**(5), 774 (2002)
8. Kulkarni, S., Mitra, S.: Management of remote mediation for children's education over the internet. In: Global Learn, pp. 2044–2049 (2010)
9. Catano, V.M.: Relation of improved performance through verbal praise to source of praise. Percept. Mot. Skills **41**(1), 71–74 (1975)

10. Sugawara, et al.: Social rewards enhance offline improvements in motor skill. PLoS ONE **7**(11), e48174 (2012)
11. Reeves, B., Nass, C.: How people treat computers, television, and new media like real people and places. CSLI Publications and Cambridge University Press, Cambridge (1996)
12. Kahn Jr., P.H., et al.: "Robovie, you'll have to go into the closet now": children's social and moral relationships with a humanoid robot. Dev. Psychol. **48**(2), 303 (2012)
13. Hayashi, K., Shiomi, M., Kanda, T., Hagita, N.: Are robots appropriate for troublesome and communicative tasks in a city environment? IEEE Trans. Auton. Ment. Dev. **4**(2), 150–160 (2012)
14. Nass, C., Steuer, J., Tauber, E.R.: Computers are social actors. In: Proceedings of the SIGCHI Conference on Human Factors in Computing Systems, pp. 72–78 (1994)
15. Johnson, D., Gardner, J., Wiles, J.: Experience as a moderator of the media equation: the impact of flattery and praise. Int. J. Hum. Comput. Stud. **61**(3), 237–258 (2004)
16. Mumm, J., Mutlu, B.: Designing motivational agents: The role of praise, social comparison, and embodiment in computer feedback. Comput. Hum. Behav. **27**(5), 1643–1650 (2011)
17. Leyzberg, D., Spaulding, S., Scassellati, B.: Personalizing robot tutors to individuals' learning differences. In: Proceedings of the 2014 ACM/IEEE International Conference on Human-Robot Interaction, pp. 423–430 (2014)
18. Warren, Z.E., Zheng, Z., Swanson, A.R., Bekele, E., Zhang, L., Crittendon, J.A., Weitlauf, A.F., Sarkar, N.: Can robotic interaction improve joint attention skills? J. Autism Dev. Disord. **45**(11), 3726–3734 (2015)
19. McGaugh, J.L.: Memory–a century of consolidation. Science **287**(5451), 248–251 (2000)
20. Walker, M.P., Stickgold, R.: Sleep-dependent learning and memory consolidation. Neuron **44**(1), 121–133 (2004)
21. Robertson, E.M., Pascual-Leone, A., Miall, R.C.: Current concepts in procedural consolidation. Nat. Rev. Neurosci. **5**(7), 576 (2004)
22. Walker, M.P.: A refined model of sleep and the time course of memory formation. Behav. Brain Sci. **28**(1), 51–64 (2005)
23. Zajonc, R.B.: Social Facilitation: Research Center for Group Dynamics. Institute for Social Research, University of Michigan, Ann Arbor (1965)
24. Karau, S.J., Williams, K.D.: Social loafing: a meta-analytic review and theoretical integration. American Psychological Association (1993)
25. Jorgensen, B.W.: Group size: its effects on group performance and subsequent individual performance (1973)
26. Thornburg, T.H.: Group size & member diversity influence on creative performance. J. Creat. Behav. **25**(4), 324–333 (1991)
27. Dennis, A.R., Williams, M.L.: A meta-analysis of group size effects in electronic. brainstorming. Emerg. E-collab. Concepts Appl. **1**, 250 (2007)
28. Sakamoto, D., et al.: Humanoid robots as a broadcasting communication medium in open public spaces. Int. J. Social Robot. **1**(2), 157–169 (2009)
29. Shiomi, M., Hagita, N.: Do synchronized multiple robots exert peer pressure? In: Proceedings of the Fourth International Conference on Human Agent Interaction, Biopolis, Singapore, pp. 27–33 (2016)
30. Iio, T., Yoshikawa, Y., Ishiguro, H.: Pre-scheduled turn-taking between robots to make conversation coherent. In: Proceedings of the Fourth International Conference on Human Agent Interaction, Biopolis, Singapore, pp. 19–25 (2016)
31. Walker, M.P., et al.: Dissociable stages of human memory consolidation and reconsolidation. Nature **425**(6958), 616–620 (2003)
32. Fischer, S., et al.: Sleep forms memory for finger skills. Proc. Natl. Acad. Sci. **99**(18), 11987–11991 (2002)
