# Supplementary material for: Two is better than one: Social rewards from two agents enhance offline improvements in motor skills more than single agent
Source: PLoS One. 2020 Nov 4;15(11):e0240622. doi: 10.1371/journal.pone.0240622 (PMC7641341; doi:10.1371/journal.pone.0240622)
Supplement: Attachment — Submitted filename: ReviewReply_PlosOne_Two_ver006.pdf [file pone.0240622.s003.pdf]

Review reply for paper
"Two is Better than One: Social Rewards from Two Agents Enhance Offline Improvements in Motor Skills More than Single Agent"

Masahiro Shiomi

We are grateful to the reviewers and editors for their detailed comments and specific feedback that enabled us to improve our paper. We considered all comments and tried to address them. We hope that the changes are satisfactory. In this document, the comments from the reviewers are underlined.

Associate Editor

Journal Requirements:
When submitting your revision, we need you to address these additional requirements.
1. Please ensure that your manuscript meets PLOS ONE's style requirements, including those for file naming. The PLOS ONE style templates can be found at https://journals.plos.org/plosone/s/file?id=wjVg/PLOSOne_formatting_sample_main_body.pdf and https://journals.plos.org/plosone/s/file?id=ba62/PLOSOne_formatting_sample_title_authors_affiliations.pdf

We modified the manuscript to follow PLOS ONE style.

2. Please include in your methods section a short description of how participants were recruited.

The participants were recruited via campus flyers at several universities in Japan. We added these information to the method section (Line 177).

3. We note that you have indicated that data from this study are available upon request. PLOS only allows data to be available upon request if there are legal or ethical restrictions on sharing data publicly. For more information on unacceptable data access restrictions, please see http://journals.plos.org/plosone/s/data-availability#loc-unacceptable-data-access-restrictions.
In your revised cover letter, please address the following prompts:
a) If there are ethical or legal restrictions on sharing a de-identified data set, please explain them in detail (e.g., data contain potentially sensitive information, data are owned by a third-party organization, etc.) and who has imposed them (e.g., an ethics committee). Please also provide contact information for a data access committee, ethics committee, or other institutional body to which data requests may be sent.
b) If there are no restrictions, please upload the minimal anonymized data set necessary to replicate your study findings as either Supporting Information files or to a stable, public repository and provide us with the relevant URLs, DOIs, or accession numbers. For a list of acceptable repositories, please see http://journals.plos.org/plosone/s/data-availability#loc-recommended-repositories.
We will update your Data Availability statement on your behalf to reflect the information you provide.

Thank you, we uploaded the minimal anonymized data set to replicate our study findings as supporting information (Line 399).

4. We noted in your submission details that a portion of your manuscript may have been presented or published elsewhere. Please clarify whether this [conference proceeding or publication] was peer-reviewed and formally published. If this work was previously peer-reviewed and published, in the cover letter please provide the reason that this work does not constitute dual publication and should be included in the current manuscript.

Thank you, we modified the cover letter about the differences of this paper and our previous trial. As described in the cover letter, a part of the initial trial is presented in a conference proceeding [1]; however, this manuscript was substantially modified by adding new conditions to the experiments with more participants compared to the published conference proceeding. In summary, the differences between the past work and this work are:

[1]:
- The number of participant: 27
- The number of condition: one (Number factor, we did not use displayed agents: 1x 3 conditions)
- Results: Two-agents condition is significantly better than no-praise condition, but comparison between two-agents and one-agent conditions and one-agent and no-praise conditions did not show significant differences) and it did not investigate the presense factor's effect

This paper:
- Participant number: 96
- Condition: two (Presence factor and number factor, 2 x 3 conditions)
- Results: The effectiveness of praises from multiple agents written in the paper, and investigated the presense factor's effects

Based on these differences, we believe that this manuscript would provide rich knowledge to readers and should have substantial differences from the past study. We added these explanations to the current manuscript (Line 67).

[1] Soto Okumura, Mitsuhiko Kimoto, Masahiro Shiomi, Takamasa Iio, Katsunori Shimohara, Norihiro Hagita, "Do Social Rewards from Robots Enhance Offline Improvements in Motor Skills?," Proceedings of the Ninth International Conference on Social Robotics, pp. 32-41, 2017.

5. Please include captions for your Supporting Information files at the end of your manuscript, and update any in-text citations to match accordingly. Please see our Supporting Information guidelines for more information: http://journals.plos.org/plosone/s/supporting-information.

We added captions for supporting information at the end of the manuscript, including the minimal anonymized dataset.

Additional Editor Comments (if provided):

The paper is sound and interesting, however it has to be improved for better clarity. Both reviewers have done excelent job in specifying what has to be done to reach perfect result. I strongly encourage You to introduce their suggestions and proceed

Thank you very much! We modified the paper based on reviewers' comments. The details of changes are described below.

Reviewer 1

The article introduces a study to show that positive feedback from virtual agents can improve offline learning. The study is timely and relevant for the field, although it suffers from a number of shortcommings that should be addressed. I detail them below.

Thank you, we modified the paper based on your review comments.

English should be improved. For example, in the abstract:
Such social rewards as praise from others enhance offline improvements in our motor skills. Does praise from artificial beings, e.g., computer-graphics-based agents (virtual

should read:

Social rewards such as praise from others…

Also, in the introduction:
Thus, the following is our first research question:
should be rephrased.

Thank you, we modified the texts .

Also:
The effects of enhancing offline improvement in motor skills resemble a kind of social influence from others. In the context of social influence, one leading factor in human-human interaction is the power of numbers; human behaviors and performance change consciously/unconsciously due to an increase of the number of people, such as social facilitation/loafing [19-23]. The power of the number effect is also observed in human-agent and humanrobot
interactions [24-26]. We hypothesize that the number of agents influences the effects of praise. Thus, the following is our second research question:

This paragraph should be rewritten almost entirely to read better. For example:
The effects of enhancing offline improvement in motor skills resemble a kind of social influence from others.
You probably mean:
Positive Social feedback is an important factor in learning, and it improves offline learning.

We modified the paragraph based on your comments.

In the disucssoin:

Still our study only…

This is again a strange construction.

I can find similar constructions all across the article, and would recommend addressing them by trying to put simpler grammatical structures, or have a native speaker thoroughly review the article.

Thank you, we modified the texts and used a commercial English proofreading service.

Regarding references:

A key concept of the paper stated in the introduction is offline consolidations (a critical process for learning) but has no reference to point to. Although this is a basic concept, since this kind of article might also be read by software engineers with no background in learning science I would advise to add relevant references for this.

Thank you, we modify the text to explain about the offline consolidations, and add two references about the basic concept, as "However, even if studies identified the effectiveness of praise, it remains unknown whether the praise from such agents has a similar effect on an offline consolidation, which is a critical learning process that is related to the amount of time spent on the learning [19, 20], as praise from humans" (Line 40).

[19] E. M. Robertson, A. Pascual-Leone, and R. C. Miall, "Current concepts in procedural consolidation," Nature reviews. Neuroscience, vol. 5, no. 7, pp. 576, 2004.
[20] M. P. Walker, "A refined model of sleep and the time course of memory formation," Behavioral and brain sciences, vol. 28, no. 1, pp. 51-64, 2005.

There are a striking number of similarities of this work with a work they cited (reference 9, which can be found here:
https://journals.plos.org/plosone/article?id=10.1371/journal.pone.0048174

Authors should better clarify in the introduction that the designed is very similar (task, variable manipulated), but changing the way the praise is given (in one case a movie, in another a robot), and that the knowledge that the agent is not a real human (in the movie participants were told these were real people giving live feedback) does not change the main challenge.

Thank you, we agree that the importance of explicitly explain the differences between our study and the past study, We added the explanations at the end of the introduction, including the explanations about the differences between this study and our past study, as "We note that this study is an extended version of a previous work [32] and added conditions to the experiments with more participants, analysis, and discussions. Moreover, this study design is following the past study that investigated praise effects toward offline improvement from people [9]. We changed the way of praising, i.e., agents praise participants instead of human experimenter's movie." (Line 67)

[9] S. K. Sugawara, S. Tanaka, S. Okazaki, K. Watanabe, and N. Sadato, "Social rewards enhance offline improvements in motor skill," PLoS ONE, vol. 7, no. 11, pp. e48174, 2012.
[32] S. Okumura, M. Kimoto, M. Shiomi, T. Iio, K. Shimohara, and N. Hagita, "Do Social Rewards from Robots Enhance Offline Improvements in Motor Skills?," in International Conference on Social Robotics, pp. 32-41, 2017.

Regarding experimental design:

The experiment is well designed, and it avoids a number of possible confounding factors. However, in the no-physical condition actually rendered a character of similar size and position than the physical

robot. It is possible to expect that an agent that is not-embodied (i.e., a voice with no body) would produce a different effect. I would therefore avoid the term non-physical, and rather opose a physical robot to a digital character.

As the review pointed out, we modified the term (non-physical) in this paper. Based on the past studiy that investigated the differences between robots and computer-graphics based robots [29], we decided to use the term of "collocated" for the robots, and the term of "displayed" for the computer-graphics based robots. We also used the word of "sence of presence" instead of physicality factor, and modified captions of graphs (e.g., (Line 17).
In addition, the reviewer's suggestion ("not-embodied (i.e., a voice with no body) would produce a different effect") is interesting point, therefore we added descrotpions into the discussion section (Line 276).

[29] A. Powers, S. Kiesler, S. Fussell, and C. Torrey, "Comparing a computer agent with a humanoid robot," in Human-Robot Interaction (HRI), 2007 2nd ACM/IEEE International Conference on, pp. 145-152, 2007.

Results.

I believe this could be improved.
Figure 2.a shows the average number of sequences completed in 30 seconds, but does not report on inter-subject variability which, I would argue, is quite important for a between group design. I would recomment including this.

The original figure includes standard deviation (S.D) to show inter-subject variability within a group, but we modified the figure to show standard error (S.E.) as an inter-subject variability between a group because the graphs of S.D are overlapped and the readability was low. Instead, we added a table to explain the average values, S.D and S.E. (Line 230).

Figure 2.b shows there is an effect of the number of agents. It also shows a trend in the impact of the physicality of the agent (a physical robot or a robot rendered on a screen).
However, the interpretation states clearly authors should also explain what the vertical axis stands for in the caption of the figure.
authors should also state clearly that there seems to be a trend towards the fact that the physicality of the agent impacted the improvement, but the analysis method chosen (anova with bonferroni correction) does not reflect that

Firstly, we added the explanations about the vertical axis to the caption of the figure, as "Rate of offline improvement (the vertical axis indicated that the percentage of the increase from the mean performance of day 1 to 2.)" (Line 237)
About the physicality effects, as the reviewer pointed out, the collocated agents seemed to be better than displayed agents. However, these effects might be weak and smaller than inter-subject variability therefore the statistical analysis did not show significant effect. In fact, the effect sizes of them also quite small. Therefore, even though the collocated agents seemed to be better than displayed agents, it would be important to clearly describe that the improvement effects are limited and the statistical analysis did not show any effects on the presence factor.
For reference, even if we only compared the experiment data without no-praise conditions or compared displayed/collocated condition separately, there is no significant difference. In addition, related to the below commets, we tested another statistical analysis with a generalized linear

model for the percentage of the increase from the mean performance of day 1 to 2. The results showed a similar trend to ANOVA's results, i.e., we found a significant difference in the number factor (p<.001) but no significant difference in the presence factor (p=0.180).
We modified descriptions about these phenomenon in the results and discussion section (Line 206, Line 258).

It is entirely possible that the election of the analysis method is affecting this conclusion. I have not seen anywhere whether the measure shown in figure 2.b is normally distributed, which should be tested before performing an ANOVA analysis.
A linear regression, a generalized linear model or a bayesian analysis might be more suited than an ANOVA for this purpose.

We agree that we need to test before performing an ANOVA analysis. The levene's test indicated that equality of error variances was assumed at the significant level of 0.05 (p=0.392), and the Kolmogorov-Smirnov test indicated that the data is consistent with the nomal distribution (p=0.200) (Line 199).
As the reviewer suggested, there are several statistical methods exist for this analysis. However, our interests in multiple comparison in the number factor, and the results of above analysis allows us to use ANOVA, therefore we would like to use the ANOVA for this analysis.
As a reference, we conducted a generalized linear model for the percentage of the increase from the mean performance of day 1 to 2. The results showed a similar trend to ANOVA's results, i.e., we found a significant difference in the number factor (p<.001) but no significant difference in the presence factor (p=0.180).

In figure 2.c
rating questionnaires are ranked variables, and therefore not suited for anova analysis. Authors should resort to non-parametric tests, or do another kind of analysis.

I would also recommend providing the anonymized dataset together with the article submission in order to facilitate a re-analysis by other teams. If they provided the code

Similar to above comments, we agree that we need to test before performing an ANOVA analysis. For the happiness rating, the levene's test indicated that equality of error variances was assumed at the significant level of 0.05 (p=0.208) but the the Kolmogorov-Smirnov test did not indicate that the data is consistent with the nomal distribution (p<.001). Moreover, for the degree of perceived praise, the levene's rest did not indicate that equality of error variances at the significant level of 0.05 (p=0.029), but the the Kolmogorov-Smirnov test did not indicate that the data is consistent with the nomal distribution (p<.001). As described above, we are interested in multiple comparison in the number factor, therefore, we performed a Box-Cox transformation for the questionnaire results. A two-way ANOVA showed similar results compared to the original results for both questionnaires.
About the happiness rating, the results showed showed a significant main effect in the number factor (F(2, 90)=25.312, p<.001, *partial* η^2=.360). No significance was found in the type factor (F(1, 90)=0.435, p=.511, *partial* η^2=.005) or the interaction effect (F(2, 90)=0.199, p=.820 *partial* η^2=.004). Multiple comparisons with the Bonferroni method revealed a significant difference: two > no-praise (p<.001) and one > no-praise (p<.001). We found no significant differences between two and one (p=1.000).
About the degree of perceived praise, the results showed a significant main effect in the number factor (F(2, 90)=168.085, p<.001, *partial* η^2=.789). No significance was found in the type factor (F(1, 90)=0.584, p=.447, *partial* η^2=.006) or the interaction effect (F(2, 90)=0.578, p=.563,

partial η^2=.013). Multiple comparisons with the Bonferroni method revealed a significant difference: two > no-praise (p<.001) and one > no-praise (p<.001).
We modified these descriptions in the results section (Line 211).

Discussion
I see a number of elements missing in the discussion that should be nuanced

Authors state: our experiment results did not show significant differences in physicality in the context of offline motor skill improvements

this can be misleading given the previous comments on the results of figure 2.b

Based on the responses for the above comments, we modified descriptions as: "On the other hand, even though our experiment results suggest that the presence factor impacted the improvement, it did not significantly affect their offline improvements, i.e., the presence effects of agents is limited in our experimental contexts." (Line 258)

-Immersive virtual reality (and recently, Immersive Augmented reality) has shown that the reaction to digital characters can be quite similar to physical agents, even with low quality of rendering. This is probably caused by the feeling of having a shared physical space with a digital agent, and therefore a social interaction much closer to real social interaction.

see, for example
https://dl.acm.org/doi/10.1145/1857893.1857896
https://journals.plos.org/plosone/article?id=10.1371/journal.pone.0032931
https://vhil.stanford.edu/pubs/2018/does-a-digital-assistant-need-a-body/

I recommend the authors to discuss this fact in the context of their experiment.

As the reviewers provided, several studies reported people's similar reactions to agents in VR/AR setttings [43-45]. Our experiment results showed that displayed agents relatively weak effects compared to collocated robots (but there is no significant differences between them), but following the related studies, immersive virtual reailty or augmented reality will increase the effects of displayed agents. Therefore, investigating the praise effects from displayed agens between physical/virtual environment would be one interesting future work (Line 276) .

[43] X. Pan, M. Gillies, C. Barker, D. M. Clark, and M. Slater, "Socially anxious and confident men interact with a forward virtual woman: an experimental study," PloS one, vol. 7, no. 4, 2012.

[44] J. Llobera, B. Spanlang, G. Ruffini, and M. Slater, "Proxemics with multiple dynamic characters in an immersive virtual environment," ACM Trans. Appl. Percept., vol. 8, no. 1, pp. Article 3, 2010.

[45] K. Kim, L. Boelling, S. Haesler, J. Bailenson, G. Bruder, and G. F. Welch, "Does a digital assistant need a body? The influence of visual embodiment and social behavior on the perception of intelligent virtual agents in AR," in 2018 IEEE International Symposium on Mixed and Augmented Reality (ISMAR), pp. 105-114, 2018.

[46] D. DeVault, R. Artstein, G. Benn, T. Dey, E. Fast, A. Gainer, K. Georgila, J. Gratch, A. Hartholt, M. Lhommet, G. Lucas, S. Marsella, F. Morbini, A. Nazarian, S. Scherer, G. Stratou, A. Suri, D. Traum, R. Wood, Y. Xu, A. Rizzo, and L.-P. Morency, "SimSensei kiosk: a virtual human interviewer for healthcare decision support," in Proceedings of the

2014 international conference on Autonomous agents and multi-agent systems, Paris, France, pp. 1061-1068, 2014.
[47] O. Rudovic, J. Lee, M. Dai, B. Schuller, and R. W. Picard, "Personalized machine learning for robot perception of affect and engagement in autism therapy," Science Robotics, vol. 3, no. 19, pp. eaao6760, 2018.

In addition to the nuancing of the impact of the physicality of the agent (and eventually, a re-analysis of that result), I would also recommend nuancing the statements regarding the number of agents. It is unlikely this effect would scale linearly. For example, whether having 4 robots, or 8 robots would improve the feedback (keeping the feedback sentences stable).

We agree that the number effects are also interesting topics for this context. This study showed that praises from two agents are better than one agent, but we did not investigate the effects of three or more robots in this study. Number of others will increase such social influences and saturation number would exist. For example, past studies reported that the number of others have influences toward social facilitation[39], social loafing[40], and peer pressure effects [41]. A past study about peer pressure effects from social robots reported that the number of robots changes their peer pressure effects, but they also discussed that total power of them are relatively weak compared to humans [42]. We thought that praise effects also have been influenced by the number of robots and might be weaker than human participants. Investigating such effects would be one interesting future works. We added these disucssions to the paper (Line 282).

[39] R. B. Zajonc, "Social facilitation," Science, vol. 149, no. 3681, pp. 269-274, 1965.
[40] S. J. Karau, and K. D. Williams, "Social loafing: A meta-analytic review and theoretical integration," Journal of personality and social psychology, vol. 65, no. 4, pp. 681, 1993.
[41] S. E. Asch, "Opinions and Social Pressure," Scientific American, vol. 193, pp. 17-26, 1955.
[42] M. Shiomi, and N. Hagita, "Do the number of robots and the participant's gender influence conformity effect from multiple robots?," Advanced Robotics, vol. 33, no. 15-16, pp. 756-763, 2019.

Structure.

In the results I can read this: Each group had 16 participants, all of whom came to our laboratory on back-to-back days. They were trained on a sequential finger-tapping task for which offline improvement has previously been described [9, 30-32]. Their performance was defined by the number of correctly tapped sequences per 30-second trials.

I believe this should be in the methods section.

Thank you, we re-organized the paper; therefore the method section was before the results section, so we deleted these texts from the results section to avoid repeating descriptions.

I also can read this:Note that the amount of praise is identical between groups; in the "Praise with one virtual agent" and "Praise with one physical agent" groups, the virtual/physical agents provided two sentences of praise. In the "Praise with two virtual agents" and "Praise with two physical agents" groups, each virtual/physical agent made just one sentence of praise.

This kind of clarification could probably be avoided if the methods section was before the results.

Thank you, as described above, we re-organized the paper; therefore the method section was before the results section, so we deleted these texts from the results section to avoid repeating descriptions.

also, in the methods sectoin I found:
By following a past study's praise manipulation

I would recommend that authors reference the study mentioned

We added the references and modified the text, as "By following past studies' praise manipulation [9][32], the agents…" (Line 119)

Reviewer 2

The paper presents one study aimed to determine whether the offline motor skills of a human partner could be improved by (1) the praise from a synthetic agent; (2) the number of agents that praise; (3) the type of physicality of the agent (virtual vs. physical).

The paper has many positive aspects (i.e., the proposed methodology is sound; the statistical treatments are adequate; Fig 1 and 2 are clear and very helpful to understand quickly the design and to picture the experiment done). The topic is relevant, and the paper could contribute significantly to the body of literature on Human Agent/Robot Interaction in general and specifically in an educational context.

However, there are some limitations (that can be fairly easily addressed) which should be addressed before publication. Although the theoretical part is short, it is direct and explicit.

Thank you very much! We modified the paper based on your review comments.

MISCELLANEOUS - MINOR ISSUES
The Fs, ps and other indicators should be in italics.
A table with the means and the standard deviations should be presented.
Some typos need to be corrected: ex: "popele would be effective moreat than praise from one person"
DOF = degree of freedom? Make it clear for the reader who is not used to with robotics.

Thank you, we modified these points. DOF is degree of freedom, we added explanations of abbreviations (Line 91).

MAJOR ISSUES
Although I do understand the initial intention of the authors, I would suggest a reorganization of the paper. Indeed, the present organization does not help to easily understand the experiment. For example, information about the experimental design, the procedure, or the material are either repeated in different sections (sometimes three times) or explained too "late" to have a big picture of the experiment that would help to understand the results (e.g., the reader has to wait the Results section to know that Happiness and Perceived Praise have been measured, as well as the way they were measured).
Consequently, I would suggest a more traditional organization to avoid repetitions: theoretical part, method with sample description, material description, procedure etc.. and then the results section and the discussion.

As suggested by the reviewer, we re-organized paper by following a traditional organization. We hope these modifications improved the redabilities of the paper.

Moreover, how happiness and perceived praise were measured is not described. It is only said that it was measured "by a questionnaire on a seven-point-scale". Instruments should be described as well as their reliability if applicable (e.g. Cronbach alpha).

We added the detail information about questionnaires in the subsection about the measurement sub section (with re-organized version): "As subjective items, we gathered two questionnaire items to investigate the feeling of the perceived happiness towards the agents' speech contents

("I felt happiness after listening at the agent speeches"), and the degree of perceived praise ("I think that the agents praised me"). For these questionnaires, we used the response format on seven-point-scale, i.e., describing the options ranging from "1:strongly disagree" to "7: strongly agree" [33]. (Line 178)"

We used a single item for each measurement, therefore we did not investigated the reliability of them. In the past version, we simply used ANOVA but we noticed that we need to additional analysis to use ANOVA (e.g., equality of error variances) for such variables based on other reviewer's comments. For the happiness rating, the levene's test indicated that equality of error variances was assumed at the significant level of 0.05, therefore we keep current descriptions, for the degree of perceived praise, the levene's rest did not indicate that equality of error variances at the significant level of 0.05. Therefore, we performed a Box-Cox transformation for the questionnaire results and the levene's test indicated that equality of error variances was assumed at the significant level of 0.05. A two-way ANOVA showed similar results compared to the original results; the results showed a significant main effect in the number factor ($F(2, 90)=168.085$, $p<.001$, *partial* $\eta^2=.789$). No significance was found in the type factor ($F(1, 90)=0.584$, $p=.447$, *partial* $\eta^2=.006$) or the interaction effect ($F(2, 90)=0.578$, $p=.563$, *partial* $\eta^2=.013$). Multiple comparisons with the Bonferroni method revealed a significant difference: two > no-praise ($p<.001$) and one > no-praise ($p<.001$) (Line 211).

<u>Practical implications of the influence of praise on offline motor skills in educational context (for example) should be explored in the discussion.</u>

Thank you, we added the discussion about the practical implications of the influence of praise on offline motor skills in educational context into the discussion section. For example, in education context, supporting basic computer skills such as inputting characters by a keyboard would be one possible application in educational context. We added the below descriptions to the discussion section (Line 251):

Offline motor skill improvement would be useful for the education support of children, e.g., learning such basic computer skills as inputting characters by a keyboard. Moreover, if the effects of praise from multiple agents improve both offline motor skills and motivation, the knowledge will be helpful for designing educational support systems because such recent systems often use displaced/collocated physical agents to support learning. In fact, several studies investigated the effectiveness for such learning support agent systems [34, 35]. Since they reported some positive effects of praise from a single collocated robot, investigating praise effects on other education topics is interesting future work.
